# The diatonic sound of scent imagery

**DOI:** 10.1177/03010066251342011

**Published:** 2025-06-03

**Authors:** Oriente Pimentel, Erick G. Chuquichambi, Charles Spence, Carlos Velasco

**Affiliations:** 385941 Basque Culinary Centre, Spain;; Centre for Multisensory Marketing, Department of Marketing, BI Norwegian Business School, Norway; Human Evolution and Cogniton group, Deparment of Psychology, University of the Balearic Islands, Spain Centre for Multisensory Marketing, Department of Marketing, BI Norwegian Business School, Norway; University of Oxford Department of Experimental Psychology, Cross-modal Research Laboratory, UK; Centre for Multisensory Marketing, Department of Marketing, BI Norwegian Business School, Norway

**Keywords:** crossmodal correspondences, smell, sound, musical modes, fragrances

## Abstract

This research investigates crossmodal correspondences between auditory stimuli, specifically musical modes, and olfactory mental imagery, represented by fragrance families. Building on the emerging literature on crossmodal correspondences, this research explores different mechanisms that might help to explain these crossmodal correspondences such as their shared connotative meaning and identity-based meaning. The first study evaluated the fragrance families and subfamilies and musical modes and assessed potential mechanisms behind these associations. The second study examined the associations between the musical modes and fragrance families and subfamilies through a matching task. The results revealed consistent matches between different musical modes and corresponding fragrance families and subfamilies, indicating a crossmodal association between auditory and olfactory mental imagery. What is more, major modes were perceived as brighter and less intense, and were more liked than minor modes, with floral and fresh fragrances similarly rated as brighter and more liked than oriental and woody fragrances. These results suggest that crossmodal correspondences between auditory and olfactory stimuli are influenced by brightness, intensity, and hedonic factors. Understanding such crossmodal associations can potentially benefit various fields, including marketing, product design, and those interested in creating multisensory experiences.

## Introduction

Human perception is a complex system that seamlessly integrates information from various sensory modalities to construct our experience of the world. Over the years, several mechanisms that influence such multisensory integration have been studied, including crossmodal correspondences, whereby people associate features, no matter whether merely imagined or physically present, across the senses (see Spence, 2011, for a review). Numerous investigations have explored how stimuli associated with different sensory modalities relate and influence one another such as olfaction and vision (Lin et al., 2018; Spence, 2020a), vision and audition (Evans & Treisman, 2011; Gallace & Spence, 2006), and taste and vision (Spence, 2023).

With respect to olfactory attributes, a series of studies examined how odours are associated with attributes from other sensory modalities, such as lightness (Kemp & Gilbert, 1997), geometrical shapes (Hanson-Vaux et al., 2013), and texture (Demattè et al., 2006). However, research on the relationship between auditory cues and olfactory perception has been comparatively limited, though increasingly studied (Spence et al., 2024). Historically, crossmodal associations between odour and pitch (Belkin et al., 1997) as well as musical notes and timbres (Crisinel & Spence, 2012a), have been documented, suggesting a deeper, yet not fully understood, interplay between audition and olfaction. For instance, a relevant study by Wesson and Wilson (2010) demonstrated that the olfactory tubercle in mice, a key brain structure that is also found in humans, exhibits selective responses not only to odours but also to auditory stimuli. Since then, growing evidence has reinforced this interplay showing that odours systematically map onto auditory features (Deroy et al., 2013). These findings support a robust yet complex integration between auditory and olfactory attributes. However, to fully understand the relationship between these attributes, a formal analysis of how they are categorized and the mechanisms involved in their matching is needed.

### Classifying Scents and the Auditory Shaping of Olfactory Imagery

Edwards’ (2008) classification of fragrances provides a structured way in which to categorize scents based on scents’ primary aromatic characteristics and the dominant olfactory components of their perceived attributes. This system groups fragrances into four families (floral, oriental, woody, and fresh) and 14 subfamilies created to help both consumers and professionals understand and describe the vast array of fragrances. Although initially developed on the basis of the creator's experience, the said classification has gained empirical support over the years. For example, Zarzo (2019) conducted a multivariate analysis of olfactory profiles for 140 perfumes using Edwards’ classification as a framework. By coding fragrances within Edwards’ four fragrance families and subcategories, Zarzo statistically analysed and mapped their sensory descriptors, revealing structured patterns that align with Edwards’ categorization.

Scent imagery is particularly relevant for the perception of a scent due to the close interaction between perception and imagery in the brain's neural mechanisms (Dijkstra et al., 2022; Spence, 2024). Research suggests a significant overlap of the same brain regions during the actual perception of a scent and the mental imagery of that scent (Gottfried & Zald, 2005; Spence, 2022; Stevenson & Case, 2005). Arshamian and Larsson (2014) emphasize that while there are similarities in how we process olfactory imagery and actual scents, individuals vary in their ability to mentally reconstruct odours, influenced by factors such as odour identity, odour interest, and perceptual experience, among others, highlighting the complexity of olfactory cognition. Additionally, Carrasco and Ridout (1993) provide a multidimensional analysis indicating that olfactory perception and imagery share common pathways, yet each also involves unique cognitive processes. Gilbert et al. (1996) further indicate that combining sensory modalities can improve the vividness and accuracy of scent imagery. For example, their experiments revealed that specific odours could evoke consistent colour characterizations, suggesting a robust crossmodal correspondence between olfaction and vision.

Olfactory expectations and perception are significantly influenced by features spanning multiple sensory modalities (Deroy et al., 2013). The distinctive character of soundscapes and music, shaped by a blend of sonic features rather than isolated auditory elements, offers a unique opportunity to examine how auditory environments can alter or intensify the perception of olfactory stimuli. This approach has been tested by exploring how soundscapes, specifically composed to evoke sweetness and dryness, could significantly impact the perceived sweetness of scents (Velasco & Spence, 2022). Similarly, Seo et al. (2013) reported that when sounds matched the scent semantically (e.g. Christmas carols with the smell of cinnamon or clove), people found the odours more enjoyable than when the sounds did not match or were neutral. Additionally, Ramos et al. (2011) created a detailed list of instrumental music, assessing it for how it relates to tastes, emotions, and levels of excitement or calmness, revealing strong links between music that felt “sweet” and positive. Altogether, the aforementioned studies highlight the potential for sound and musical attributes to play a pivotal role in the multisensory perception of scent. These findings also resonate with earlier research on the perception of meaning and higher-level percepts in music, which demonstrated that certain musical attributes consistently evoke specific emotional and conceptual associations (Watt & Ash, 1998; Watt & Quinn, 2007).

### Musical Modes in the Context of Crossmodal Correspondences

The present study introduces musical modes as an interesting class of auditory (musical) stimuli for consideration in crossmodal correspondences research. Musical modes, also known as diatonic modes, are fundamentally scales consisting of sequences of pitches arranged in specific orders, each with a fixed set of seven tones (C, D, E, F, G, A, B). Each of these sequences—Ionian, Dorian, Phrygian, Lydian, Mixolydian, Aeolian, and Locrian—carries with it a unique emotional quality, due to their distinct interval structures (Ramos et al., 2011; Straehley & Loebach, 2014). In the context of Western classical music from around 1600 to 1900, known as common-practice music, composers used scales such as the major scale, corresponding to the Ionian mode, and the natural minor scale, corresponding to the Aeolian mode. This period solidified the use of the tonal system for composition which remains the foundation of most Western music. Collier and Hubbard (2004) found a relationship between how people perceive the darkness or lightness of musical modes and the sizes of the intervals from the tonic to the other pitches within a mode. Further research has explored crossmodal associations between musical features and taste perception, showing that different scales and chords can evoke specific taste associations (Taitz et al., 2019). Similarly, studies on ancient Greek modes have found that the associations between specific musical modes and taste perception can be influenced by culinary education, particularly for sweet and bitter tastes (Ozilgen, 2021).

**Figure 1.**
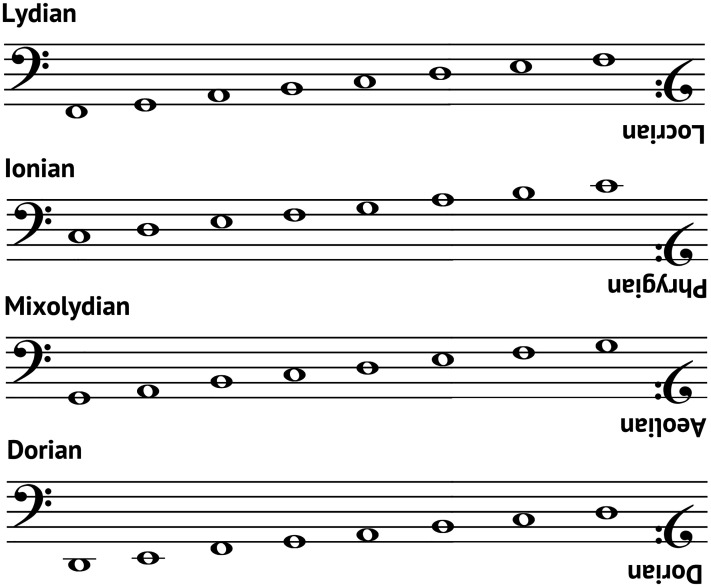
Inversion correspondence between the musical modes.

*Note*. These inversions illustrate the symmetrical properties of the diatonic system, highlighting the intrinsic relationships between different modes. When inverted through simple contrary motion, the Ionian mode (major) becomes the Phrygian mode, the Lydian becomes Locrian, the Mixolydian becomes Aeolian, while the Dorian mode remains unchanged

The concept of inversion adds another layer to our understanding of musical modes. Essentially, inversion involves flipping the sequence of intervals in a mode, creating a mirror image of the original, with each mode having a corresponding inverse, except for Dorian (Vincent, 1951). For example, in a seminal study, Serre (1753) noted that the Ionian mode (major) becomes the Phrygian mode upon inversion, while the Dorian mode remains unchanged. This symmetry, explored by theorists like Ziehn (1912), reveals that each mode has a corresponding inverse, such as Lydian inverting to Locrian and Mixolydian to Aeolian. The mathematical symmetry inherent in mode inversion ensures that each mode shares structural features with its inverse (see Figure 1). This shared structure could, therefore, lead to similar patterns in how these modes are perceived in other sensory modalities, including olfaction.

Rosar (1994) situates musical modes within a tradition of physiognomic perception, tracing their expressive function to the ancient Greek concept of ethos, where each mode embodied distinct character traits. Rather than viewing these expressions as associative or subjective emotional responses, Rosar, drawing on Werner’s theory (1978), argues that modes possess intrinsic, perceptually accessible expressive qualities. These qualities, akin to facial expressions, are directly perceived as part of the music's physiognomy, thus offering an alternative to emotion-based or associationist accounts of musical meaning. Just as musical modes can be ranked from clarity to obscurity based on their interval structure (Collier & Hubbard, 2004), scents too can be evaluated in terms of their brightness (Marks, 1978). Gilbert et al. (1996) suggested a relationship between the perceived intensity of an odour and its associated colour lightness, pointing towards a sensory dimension that integrates olfactory and visual stimuli. These authors showed that untrained participants could align auditory pitches with odours based purely on their perceptual qualities, indicating an intricate, nonhedonic aspect of odour quality influencing these matches. The underlying dimensions that define these odour qualities remained elusive but could be closely tied to constructs such as dull–aromatic, heavy–light, and bright–dark.

### The Present Study

Building on research on crossmodal correspondences, the present study assessed the extent to which the relationship between scent and musical mode might be driven by hedonic and lexical factors, and/or semantic congruency (e.g. Motoki et al., 2024). In Experiment 1, musical modes and imagery of fragrances were evaluated in terms of liking, intensity, and brightness, and using multiple semantic scales (Osgood et al., 1957) as well as an open-ended question. In Experiment 2, participants paired the musical modes and fragrances in a matching task to evaluate the potential associations between these stimuli. As proposed by Spence (2011), the psychological mechanisms explaining crossmodal correspondences are unlikely to be mutually exclusive and multiple accounts may contribute to explaining a given correspondence, thus creating a complex web of sensory interrelations in language (see also Alvarado et al., in press). But how is it that these mechanisms may underlie the relationship between musical modes and scents?

With respect to the affective mechanism, it is recognized that humans exhibit a tendency to associate stimuli from different senses based on similar levels of liking (Crisinel & Spence, 2012b; Schifferstein & Tanudjaja, 2004; Seo & Hummel, 2011; Spence, 2020a; Velasco et al., 2015). This suggests that a positive hedonic response in one sensory domain can lead to an association with another sensory domain that evokes a comparable liking response (Zhou & Yamanaka, 2018).

Furthermore, the current study leverages the notion of “brightness” as a key metric for assessing linguistic associations, drawing upon the foundational work of Erich von Hornbostel (1938) who suggested brightness as a universal sensory dimension. This principle posits that the characteristic of brightness transcends individual sensory modalities, manifesting equivalently across sound, scent, and even visual and tactile perceptions (Marks, 1978). However, in a recent study, Spence and Di Stefano (2024) challenge the notion of brightness as a supramodal sensory dimension. These authors suggest that while crossmodal correspondences undoubtedly do exist, the concept of brightness does not apply uniformly across the senses. Therefore, they proposed that sensory experiences are more accurately explained by specific crossmodal correspondences rather than by universal sensory dimensions.

Lastly, semantic congruency explores meaning and identity that can bridge different sensory modalities (Chen & Spence, 2010; Laurenti et al., 2004). A piece of music might evoke specific scenes, which could then be linked to a scent carrying similar meaning or identity. These connections, rooted in associative learning (see Di Stefano et al., 2024), emphasize that sensory stimuli are encoded and recalled not just in isolation but as part of a broader, interconnected network of associations (Laurienti et al., 2004).

## Experiment 1: Assessing Meaning Overlap of Musical Modes and Fragrance Imagery

### Methods

#### Participants

One hundred and twenty-two participants were initially recruited via Prolific Academic (https://app.prolific.com/) in exchange for a payment of £8.99 per hour. Twenty-one participants were excluded because of incomplete data, technical issues, or not completing the task as instructed as commonly observed in online studies (Uittenhove, 2023). Therefore, a final sample of 101 participants (*M* age = 42.2 years, *SD* = 14.7, age range = 18–81 years, 50 female individuals, 50 male individuals, and 1 nonbinary) took part in the first experiment. This sample size is larger than previous studies on auditory–olfactory crossmodal associations that relied on actual smells (ranging from 25 to 46 participants; Crisinel et al., 2013; Crisinel & Spence, 2012a), while also accounting for the use of mental imagery and a more diverse stimulus set. All participants were based in the UK and agreed to take part in the study after reading a standard consent form. In terms of musical expertise, the distribution among participants was diverse: 7.92% held a bachelor, postgraduate, masters, or PhD degree in music; 4.95% had elementary musical education (music school); a majority, 86.14%, reported general education (primary and secondary) with no formal music training; and a small portion, 0.99%, had professional musical education (conservatory). Regarding fragrance expertise, most of the participants, 97.03%, indicated that they enjoyed fragrances but had not pursued any kind of formal education or training in this area, while 2.97% had participated in workshops, short courses, or self-guided learning about the world of fragrance.

#### Apparatus and Materials

**Figure 2.**
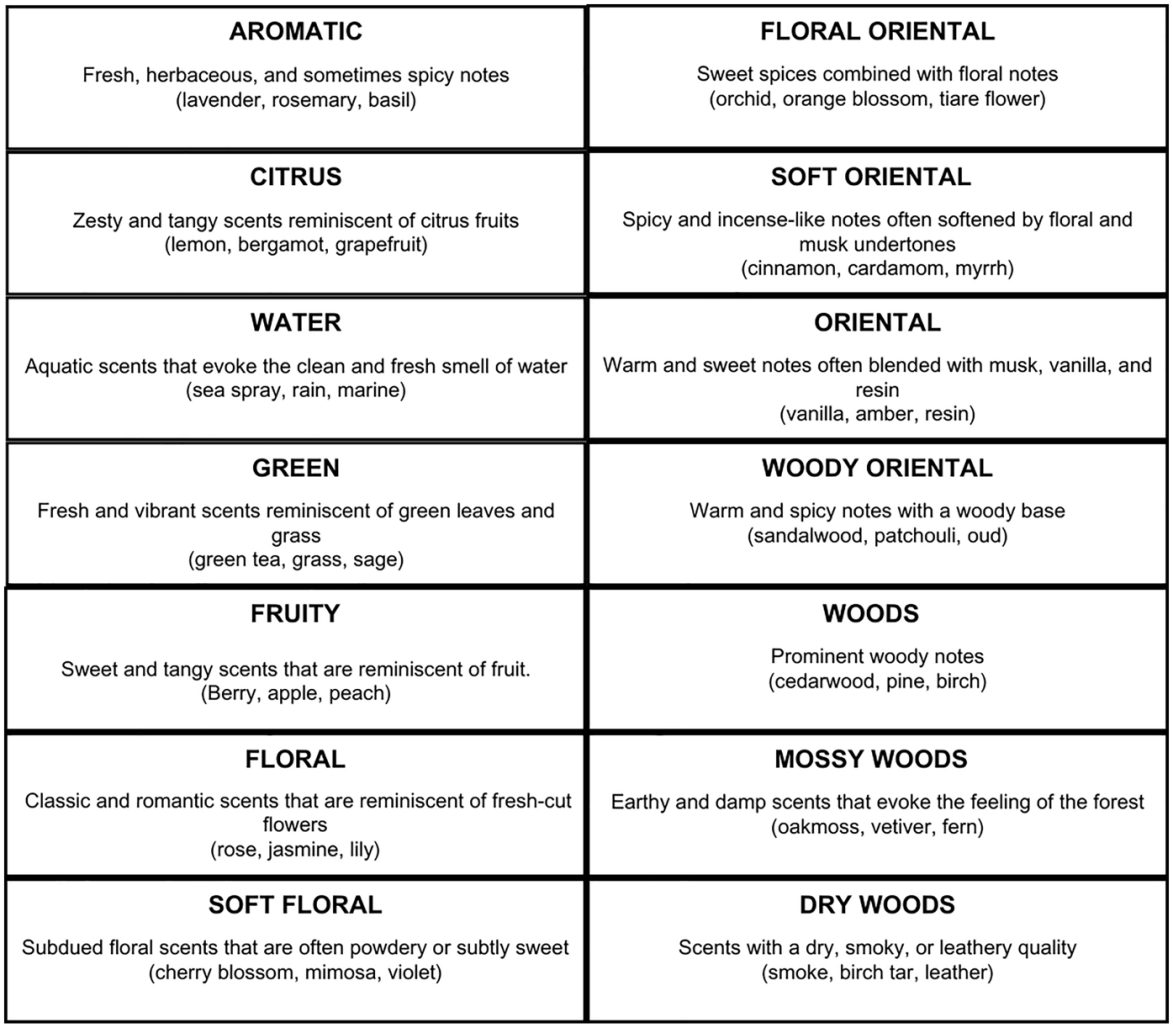
Fragrance subfamilies following Edwards’ (2008) classification.

*Note*. The 14 fragrance subfamilies, providing brief descriptions and examples.

The scent stimuli consisted of the conceptual description of the 14 different fragrance subfamilies as classified by Michael Edwards’ Wheel of Fragrances (see Figure 2) (Edwards, 2008). The wheel's design, initially based on experiential insights rather than experimental data, has been substantiated by additional research which supports its classification accuracy (Zarzo & Stanton, 2009). Further validation demonstrates the wheel's alignment with empirical data from principal component analyses (PCA) of perfume descriptions. This congruence confirms the wheel's efficacy not only in categorizing scents but also in reflecting perceptual variations that are crucial for both consumers and creators within the fragrance industry (Zarzo, 2019).

For the creation of the auditory stimuli, a single melody was adapted into the seven different musical modes: Ionian, Dorian, Phrygian, Lydian, Mixolydian, Aeolian, and Locrian. To ensure uniformity across all samples, the melody was composed in a natural key structure. Each mode variation of the melody began on the note that corresponds to the respective mode to accurately reflect its unique scale and tonal characteristics. The same musical instrument (keyboard), tone, and tempo were used for each mode to maintain consistency in the auditory stimuli, ensuring that any perceptual differences observed in the responses can be attributed to the modal variations themselves. Additionally, all samples were carefully normalized to ensure equal loudness levels, preventing any differences in volume from influencing the participants’ responses.

#### Procedure

The experiment was conducted online and designed through Qualtrics software (https://www.qualtrics.com/). Prior to participation, participants read and consented to a detailed informed consent statement that outlined the study's goals, participant rights, the anonymity of responses, and withdrawal procedures, along with contact information for further enquiries. To ensure that the participants could hear properly, a sound check was conducted where they were asked to listen to and identify a specific word in an audio track. Following this, demographic data was collected, including age, gender, and background knowledge in music and fragrances, to help categorize responses based on participants’ levels of expertise.

The core of the experiment involved exposure to two different types of stimuli. Participants read a description of each fragrance subfamily and were asked to rate them in terms of brightness, liking, and intensity using a scale from 0 to 10. Next, they rated the fragrances using the semantic differential technique (SDT), a quantitative method in which the connotative meaning of objects and concepts is measured by using rating scales with bipolar adjectives (Osgood et al., 1957). The scales consisted of 12 pairs of bipolar adjectives (awful–nice, bad–good, dead–alive, light–heavy, mild–harsh, passive–active, powerless–powerful, quiet–noisy, sad–happy, shallow–deep, slow–fast, weak–strong) previously used in studies assessing crossmodal correspondences (e.g. Velasco et al., 2016). With respect to the auditory stimuli, participants listened to the melody adapted into the seven music modes and rated each one using the same set of measures (i.e. brightness, liking, intensity, and SDT). Finally, for each fragrance and musical mode, participants reported their thoughts on the stimuli, providing qualitative data on their sensory associations. The fragrances and musical modes were presented in separate blocks. The sequence of blocks and trials was randomized. The entire process took a median of 29 min to complete.

#### Analyses

The analyses were conducted within the R environment for statistical computing (version 4.4.2, R Core Team, 2024). Likeability, brightness, and intensity ratings were analysed through one-way analyses of variance or the nonparametric Kruskal–Wallis test according to the distribution of the data. For the fragrances, the families (fresh, floral, woody, and oriental) and subfamilies (aromatic, citrus, dry wood, floral, floral oriental, fruity, green, mossy woods, oriental, soft floral, soft oriental, water, wood, and woody oriental) were analysed as within-subject factors. For the musical stimuli, the musical modes (Ionian, Dorian, Phrygian, Lydian, Mixolydian, Aeolian, and Locrian) were analysed as a within-subject factor. Dunn's posthoc tests with Bonferroni correction were conducted to identify specific pairwise differences where significant main effects were found.

SDT (Osgood, 1964) is a quantitative method used in this case to explore the semantic space that ties together the concepts of musical modes and fragrances. Following the SDT evaluations, multifactor analysis and PCA were conducted using the FactoMineR package (Lê et al. 2008) to highlight the primary dimensions of variation in the data. These techniques helped to reduce the complexity of the data by identifying key components that explain the majority of the variance, providing a clearer picture of how participants perceive and relate the different stimuli.

The qualitative data related to the participants’ thoughts on each stimulus were first screened in order to remove incomplete or irrelevant responses (e.g. vague statements such as “similar to the previous piece” or “nothing”), ensuring only pertinent data remained. An inductive, semantic-level thematic analysis (Braun & Clarke, 2006) was then conducted. Key words and phrases were identified and grouped into broader themes, for example, terms like “*strong*” and “*bold*” were categorized under *Intensity*, while “*airy*” and “*light*” were grouped under *Freshness*. This structured approach allowed for a clearer interpretation of participants’ explicit associations. Finally, a thematic frequency analysis was conducted to calculate the percentage of theme presence for each stimulus, creating a normalized measure of theme prominence.

Hierarchical cluster analysis was performed on the results of both the SDT and the thematic analysis to identify potential groupings among the fragrance subfamilies and musical modes. This analysis used Ward's method with squared Euclidean distances to ensure accurate and meaningful groupings, validated through additional statistical methods, including the silhouette method and gap statistic (Murtagh & Legendre, 2014). The “hclust” function in R was used for hierarchical clustering, while the “cluster” package was used to calculate the silhouette method (Maechler, 2019). Cluster visualizations were produced using the “factoextra” package (Kassambara & Mundt, 2020). For both the SDT and thematic analysis, similarity matrices were created to quantify the degree of similarity between each fragrance and musical mode. The SDT similarity matrix was based on the attribute ratings of the stimuli and the thematic frequency similarity matrix was based on the percentage of theme presence for each stimulus. The “pheatmap” package in R was used to visualize these matrices through two different types of clustered heatmaps (Kolde, 2019), with additional graphical elements created using ggplot2 (Wickham, 2024): one which provided a visualization of how themes and attributes were distributed across the different stimuli and the other showing which fragrance subfamily was most associated with which musical mode. The data sets and materials required for the evaluation and reproduction of the results have been made publicly available online at Open Science Framework (https://osf.io/k3jr6/).

### Results

#### Brightness, Liking, and Intensity

**Figure 3.**
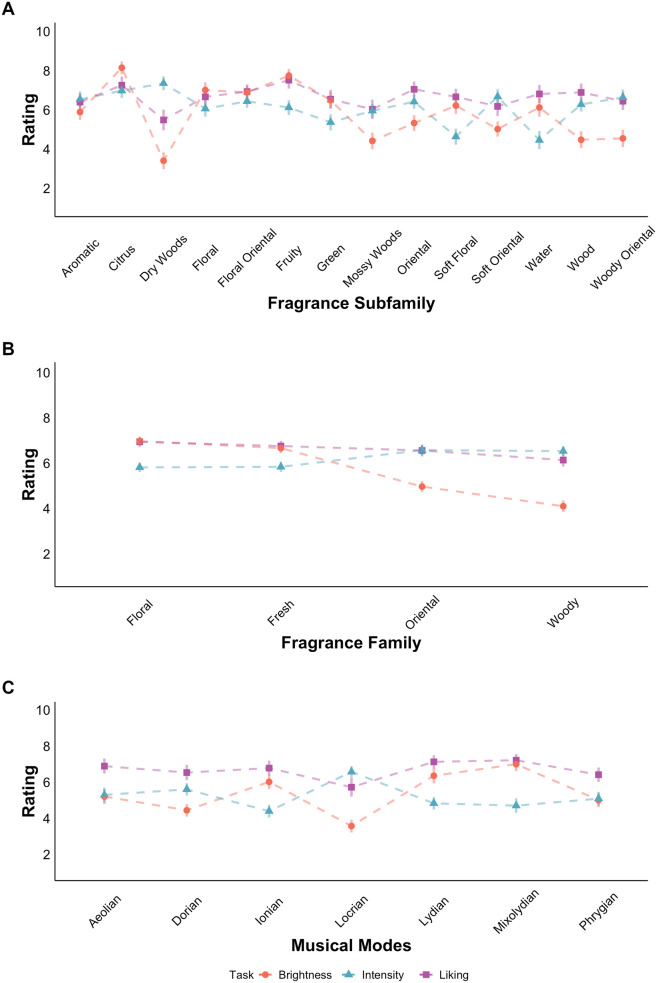
Mean ratings of likeability (square), brightness (circle), and intensity (triangle) for fragrance subfamilies (A), families (B), and musical modes (C) in Experiment 1.

*Note*. Each point on the line graphs represents the mean rating for a specific category. Error bars represent 95% confidence intervals around the means.

A summary of the results associated with the brightness, liking, and intensity ratings, for both fragrances and musical modes are presented in Table 1 (see Figure 3 for a visual summary of the results). Descriptive statistics are also provided in the Supplementary material (Table S1).

**Table 1.** Statistical outcomes for likeability, brightness, and intensity across fragrance and music categories in Experiment 1.

	Liking	Brightness	Intensity
Fragrance subfamilies			
Test	Kruskal–Wallis	Kruskal–Wallis	Kruskal–Wallis
Statistic	χ^2^(13) = 63.62	χ^2^(13) = 416.79	χ^2^(13) = 184.01
*p* value	<.001**	<.001**	<.001**
Effect size	0.045	0.295	0.045
Fragrance families			
Test	ANOVA	ANOVA	ANOVA
Statistic	*F*(3, 400) = 4.17	*F*(3, 400) = 102.40	*F*(3, 400) = 12.19
*p* value	.006*	<.001**	<.001**
Effect size	0.030	0.434	0.083
Music modes			
Test	Kruskal–Wallis	ANOVA	ANOVA
Statistic	χ^2^(6) = 27.79	*F*(6, 700) = 35.61	*F*(6, 700) = 15.12
*p* value	.001**	<.001**	<.001**
Effect size	0.039	0.234	0.115

*Note*. The Kruskal–Wallis test was used instead of analysis of variance (ANOVA) when the data did not meet the normality assumption. This table lists the specific statistical test used, the value of the test statistic, the *p*-value and the effect size. For ANOVAs, the effect size is reported as eta-squared (η^2^). For the Kruskal–Wallis tests, the effect size is reported as epsilon-squared (ε²).

*p < .01; **p < .001.

As shown in Figure 3, significant differences were found in brightness and intensity across fragrance families (B) and subfamilies (A), while liking did not differ significantly. Floral and fresh fragrances were rated as significantly brighter than woody and oriental families (*p* < .01), and woody was perceived as the most intense, differing significantly from floral and fresh (*p* < .05). These patterns were consistent across fragrance subfamilies. For musical modes (C), Lydian and Ionian were significantly brighter than Locrian and Phrygian (*p* < .01). Locrian was rated as significantly more intense than Ionian (*p* < .05). In terms of liking, Dunn's posthoc tests confirmed that Locrian was significantly less liked than Aeolian (*p* = .021), Ionian (*p* = .040), Lydian (*p* < .001), and Mixolydian (*p* < .001), while Phrygian did not significantly differ from other modes.

#### Underlying Meaning of Musical Modes and Fragrances via the Semantic Differential Technique

**Figure 4.**
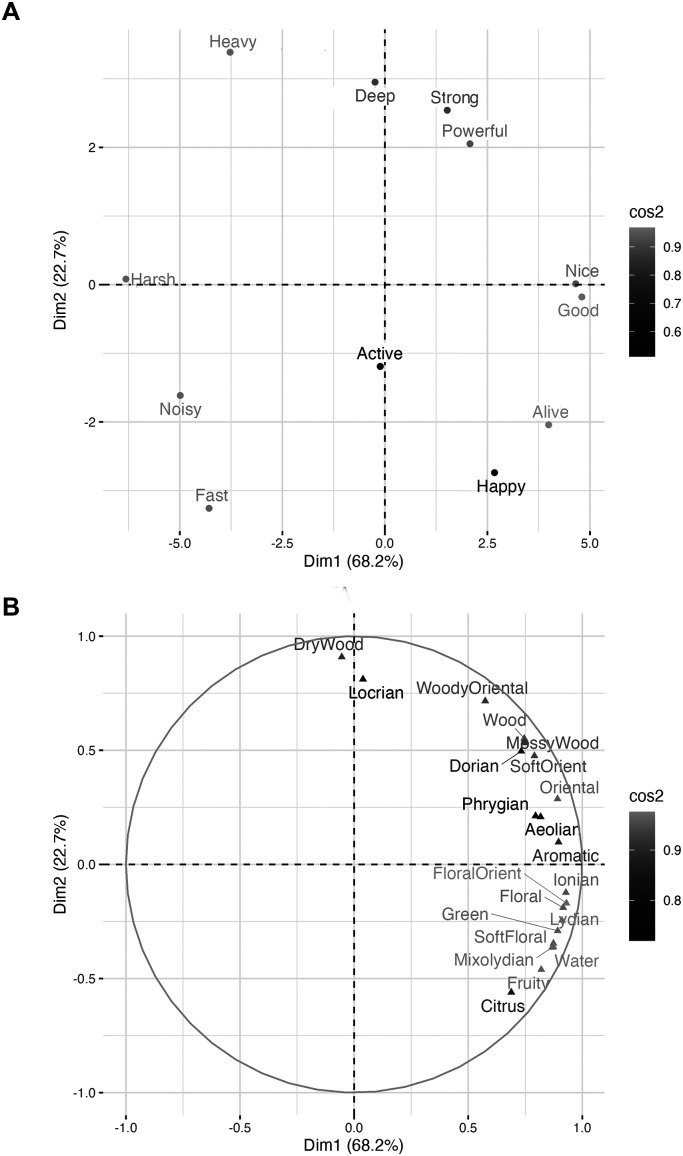
(A) Unrotated factor map of the polar scales. Note that only the label of the upper end of the scales is presented. (B) Unrotated factor map for the fragrance subfamilies and musical modes.

*Note*. Panel A shows the factor map for the bipolar adjective scales used in the study while panel B shows the factor map for the stimuli (musical modes and fragrance subfamilies). Each adjective pair and stimulus are plotted based on its loadings on the first two principal components (Dim1 and Dim2), which together explain a significant portion of the variance in the data. The grayscale shading (cos2) represents the squared cosine of the variable, indicating the quality of representation on the factor map.

The semantic differential analysis and the results of the multifactor analysis and PCA (see Figure 4) identified three significant components with eigenvalues exceeding Kaiser's criterion of 1 (Table 2). These components explained 93.2% of the variance. Table 2 shows the factor loadings after applying a varimax (orthogonal) rotation to achieve a simpler and more interpretable factor structure. This rotation maximizes the sum of the variance of the squared loadings, clarifying which variables strongly contribute to each component.

**Table 2.** Varimax-rotated component matrix in Experiment 1.

Adjectives	Component 1	Component 2	Component 3
Awful–nice	−0.120	**0**.**980**	0.060
Bad–good	−0.352	**0**.**923**	−0.100
Dead–alive	**−0**.**794**	**0**.**507**	0.180
Light–heavy	**0**.**710**	−0.473	−0.493
Mild–harsh	0.240	**−0**.**924**	−0.150
Passive–active	−0.403	0.060	**0**.**846**
Powerless–powerful	**0**.**754**	−0.387	−0.400
Quiet–noisy	0.160	−0.140	**0**.**944**
Sad–happy	**−0**.**953**	0.070	−0.230
Shallow–deep	**0**.**876**	−0.180	−0.333
Slow–fast	−0.372	0.340	**0**.**842**
Weak–strong	**0**.**684**	**−0**.**504**	−0.377
Eigenvalues	4.369	3.723	3.091
% of Variance	36.4	31.0	25.8

*Note*. The absolute value of the loading indicates the strength of the relationship between the variable and the component. Factor loadings with higher absolute values are highlighted in bold.

**Figure 5.**
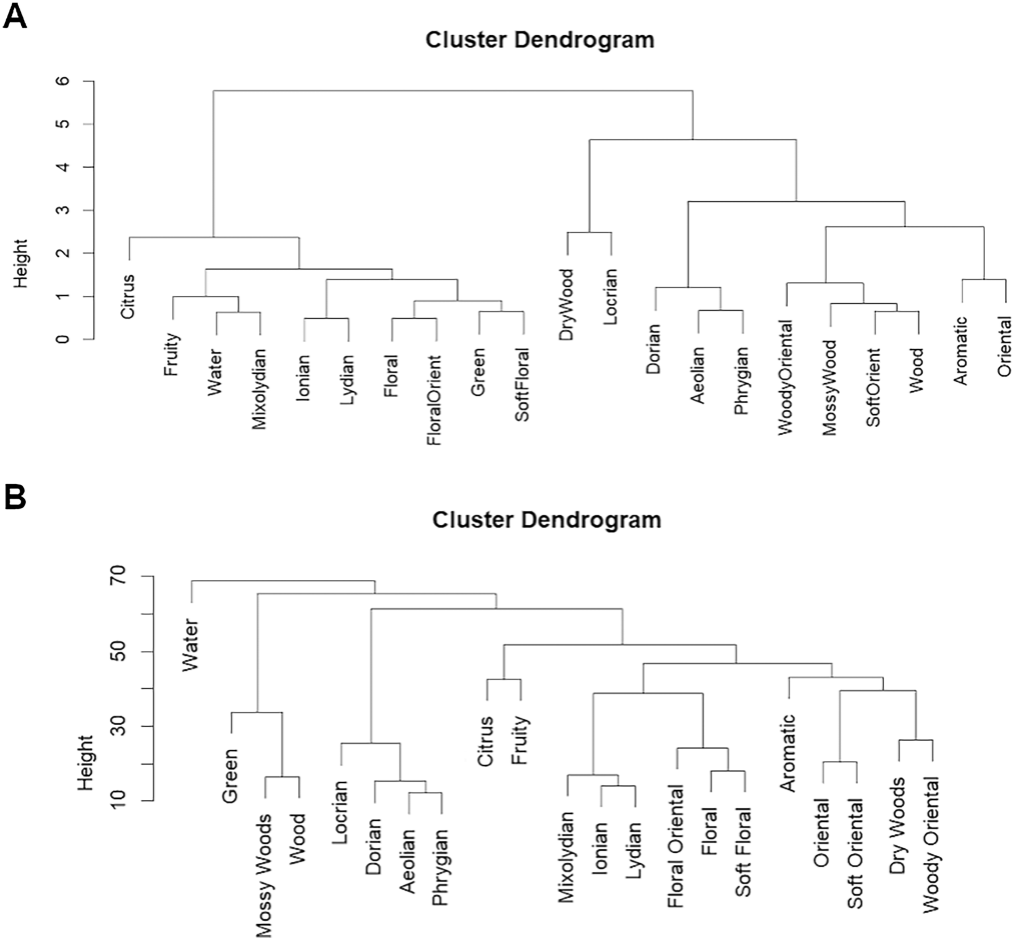
Dendrograms obtained by hierarchical cluster analyses in Experiment 1: (A) based on semantic differential technique (SDT) ratings and (B) based on thematic frequency analysis.

*Note*. In both dendrograms, height indicates the level of dissimilarity; lower heights represent higher similarity between stimuli.

The dendrogram resulting from the hierarchical cluster analysis, as shown in Figure 5A, illustrates the formation of two main clusters. The first cluster encompasses musical modes such as “Mixolydian,” “Lydian,” and “Ionian” with fragrances of the “Fresh” and “Floral” families. This grouping corresponds to the stimuli that were rated as most liked, brighter, and less intense. The second cluster stimuli were rated as more intense and less bright, includes modes such as “Dorian,” “Aeolian,” and “Phrygian” and aligns with fragrances carrying a heavier scent profile from the woody and oriental families.

#### Thematic Analysis of Musical Modes and Fragrances

Regarding the thematic analysis, a one-way analysis of variance revealed a significant main effect of the themes mentioned across different stimuli, *F*(20, 339) = 3.404, *p* < .001 η_p_^2^ = .17. This indicates that the type of stimulus significantly affects the frequency of the thematic responses from participants. The hierarchical cluster analysis (Figure 5B) also revealed a different alignment of musical modes with fragrance families than the previous attribute-based cluster. This analysis underscores a pattern whereby brighter scents from the fresh and floral families continue to align with Mixolydian, Lydian, and Ionian modes. On the other hand, these same modes are positioned closer to the oriental family. Conversely, the musical modes typically associated with intensity and a darker auditory profile—Locrian, Dorian, Aeolian, and Phrygian—remain linked with the woody family, though to a lesser extent than observed previously.

**Figure 6.**
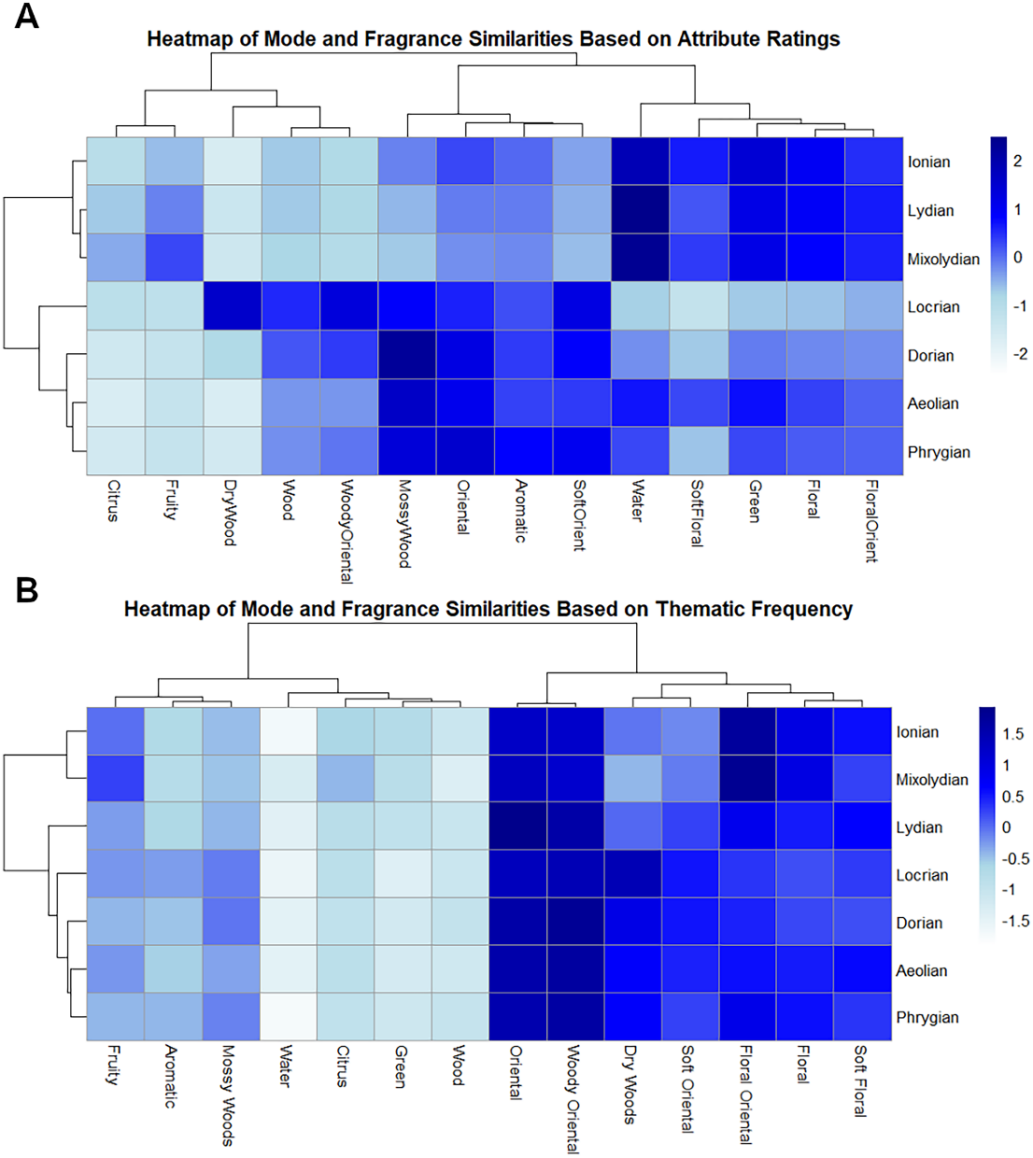
Heatmap of mode and fragrance similarities in Experiment 1: (A) similarities based on attribute ratings and (B) similarities based on thematic frequency.

*Note*. Colour intensity represents the degree of similarity between the musical modes and fragrance families. Darker blues suggest a higher degree of similarity, while lighter colours suggest a lower degree of similarity.

To visualize the difference between both semantic responses (see Figure 6), two similarity matrices were created to quantitatively assess the relationships between different fragrance families and musical modes based on the ratings of attributes and the frequency of thematic mentions in each stimulus.

Finally, qualitative results focusing on attribute ratings revealed that musical modes like Mixolydian, Lydian, and Ionian are consistently aligned with the floral and fresh fragrance families (Figure 6A). In contrast, these same modes are closer regarding thematic frequency to floral and oriental fragrances and not with the fresh fragrances (Figure 6B). Regarding minor modes, they are clustered together in both heatmaps and are also aligned with both woody and oriental fragrances. Similarities based on thematic frequency reveal two very separated clusters, one combining floral and oriental families (except for “dry woods”) and the other combining fresh and woody families (Figure 6B). This distinction is also seen with the attribute ratings with slight differences such as “water” and “green” being clustered with floral fragrances and “mossy woods” also being clustered with oriental fragrances (Figure 6A).

### Discussion

The results of Experiment 1 revealed a number of intriguing patterns in the evaluation of fragrances and musical modes, suggesting possible underlying mechanisms of sensory perception. Major modes (Mixolydian, Lydian, and Ionian) were generally perceived as brighter and less intense, while minor modes (Aeolian, Dorian, Phrygian, and Locrian) were perceived as less bright and more intense. In terms of liking, only the darkest and most dissonant modes (Phrygian and Locrian) were liked significantly less. For fragrances, floral and fresh families received higher likeability ratings compared to oriental and woody families, with floral and fresh fragrances also being rated as brighter and less intense. In contrast, oriental and woody fragrances were perceived as more intense. These convergent patterns of results suggest that liking, intensity, and brightness dimensions contribute to the mapping between musical modes and fragrance profiles. Together, our results extend previous evidence of robust crossmodal correspondences between auditory and olfactory stimuli (Spence & Deroy, 2013).

The results of the SDT identified three significant components that explained 93.2% of the variance in the data. These components reflected a combination of different sensory attributes. Furthermore, hierarchical cluster analysis revealed distinct clusters: brighter and more liked modes (Ionian, Lydian, Mixolydian) were frequently paired with floral and fresh fragrance subfamilies, while darker and more intense modes (Dorian, Aeolian, Phrygian) were associated with woody and oriental fragrances.

The thematic analysis revealed significant differences in the frequency of themes mentioned across stimuli. This result reinforces the patterns observed in the semantic differential analysis, suggesting that the participants’ sensory associations could be influenced by an interplay between multiple sensory attributes, rather than a single dimension. Together, the findings of this study indicate that certain musical modes are consistently associated with specific fragrance subfamilies, supporting the idea that brightness, hedonic, and semantic mechanisms could play significant roles in crossmodal correspondences between auditory and olfactory stimuli.

However, the results of this experiment come with some limitations. For instance, participants’ familiarity with specific musical modes or fragrance descriptors could have shaped their responses. Those with prior musical training might have recognized and associated certain modes based on their theoretical knowledge rather than purely perceptual attributes, while those with extensive experience in perfumery might have relied on industry-standard fragrance classifications rather than intrinsic olfactory qualities. Moreover, while the SDT effectively captures key perceptual dimensions, it may also introduce what is known as verbal overshadowing (Schooler & Engstler-Schooler, 1990)—a cognitive effect in which verbal descriptions interfere with nonverbal perceptual memory. In other words, when participants are asked to articulate their sensory experiences using predefined linguistic scales, they may rely on conceptual knowledge rather than direct perceptual impressions, potentially distorting their genuine sensory associations. Hence, a second experiment was conducted to address these limitations and further clarify the role of brightness, hedonic value, and semantic factors as potential underlying mechanisms of auditory–olfactory crossmodal correspondences. Specifically, Experiment 2 incorporates a direct matching task designed to explore how participants pair specific modes with fragrance subfamilies.

## Experiment 2: Matching Task of Musical Modes and Fragrance Imagery

Experiment 2 evaluated how each of the seven diatonic modes might match with the four main fragrance families and 14 subfamilies. Building on previous literature and the results of Experiment 1, we expect that “brighter” modes (e.g. Ionian, Mixolydian) would align more with lighter, floral, or fresh scents, while “darker” modes (e.g. Aeolian, Phrygian, Locrian) might evoke deeper, oriental, or woody accords. Additionally, “in-between” modes (e.g. Dorian) could show more mixed associations, bridging both floral and woody/oriental families.

### Methods

#### Participants

One hundred and thirty-six participants were initially recruited via Prolific Academic (https://app.prolific.com/) in exchange for a payment of £9.13 per hour. Sixteen participants were excluded due to incomplete data, technical issues, or not completing the task as instructed. Therefore, a final sample of 120 participants (*M* age = 43.0 years, *SD* = 13.2, age range = 18–77 years, 83 female individuals, 39 male individuals, 2 nonbinary, and 1 participant who preferred not to say) took part in the second experiment. All participants were based in the UK and agreed to take part in the study after reading a standard consent form. None of these participants took part in Experiment 1.

Regarding musical expertise, 8.8% of the participants held a bachelor, postgraduate, masters, or PhD degree in music; 11.2% had elementary musical education (music school); 77.6% reported general education (primary and secondary) with no formal music training; and 2.4% had professional musical education (conservatory). Regarding fragrance expertise, most of the participants, 93.6%, indicated that they enjoyed fragrances but had not pursued any kind of formal education or training in this area, while 5.6% had participated in workshops, short courses, or self-guided learning about the world of fragrance, and 0.8% had completed formal courses or training in perfumery.

#### Apparatus and Materials

**Figure 7.**
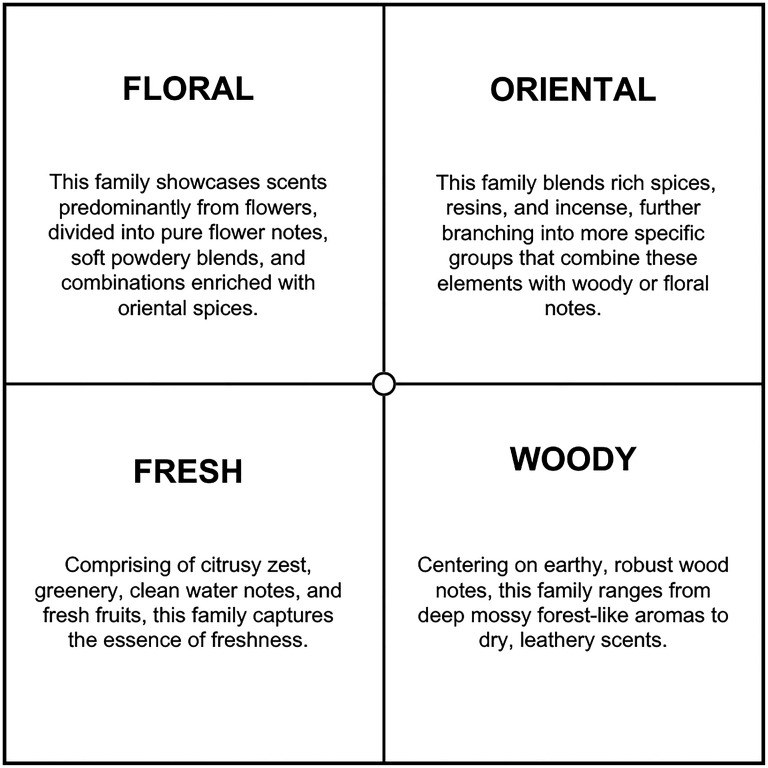
Fragrance family descriptions.

*Note*. These images present the four fragrance families, providing brief descriptions of the subfamilies in each one.

Fragrance stimuli consisted of the conceptual description of fragrance subfamilies used in Experiment 1. These descriptions also included imagery representing each of the four fragrance families, providing brief descriptions of each fragrance family to enhance the participant's ability to make accurate and meaningful associations (see Figure 7).

Regarding the sound stimuli, a different melody was created and adapted in the same way as in the first study, ensuring that each musical mode was represented accurately while maintaining consistency in instrument, tone, tempo, and loudness across all samples.

#### Procedure

As in Experiment 1, participants read and consented to a detailed informed consent statement before taking part in the experiment. A sound check was also conducted to ensure that the audio systems were functioning properly. Next, demographic data was collected, including age, gender, and background knowledge in music and fragrances, as well as self-reported hearing and olfactory impairments.

The core of the experiment involved participants listening to each of the seven musical modes and then associating each sound sample with one of the four fragrance families presented descriptively. The participants were then asked to identify which specific subfamily within the selected family they associated most with the sound. This process was repeated for each of the seven musical modes. The trial sequence was randomized. The experiment took approximately seven minutes to complete.

#### Analyses

Data were analysed within the R environment for statistical computing (version 4.4.2, R Core Team, 2024). The analyses focused on identifying the associations between fragrance families and subfamilies and specific musical modes. One participant reported having some form of smell impairment, and three participants reported having some form of hearing impairment. Sensitivity analyses revealed that the responses of these participants were not extreme values, and their exclusion had no significant impact on the results. Therefore, all participants were retained for subsequent analyses. First, a chi-square test was conducted to examine the association between musical modes and fragrance families. That is, whether the frequency distribution of fragrance families varied significantly across different musical modes. The test was executed using the “chisq.test” function from the “stats” package (R Core Team, 2024). Second, one-sample *t*-tests were used to compare participant's selections of fragrance families and subfamilies against chance level (in this case 25%). Cohen's *d* was also estimated for significant effects using the “t.test” function and “cohen.d” from the “effsize” package (Torchiano, 2020).

Additionally, the proportions of selections for all fragrance subfamilies were calculated, independent of the initial family choice. This was particularly relevant because some fragrance subfamilies, like floral oriental and woody oriental, are present in more than one family. Afterwards, McNemar tests were conducted to identify which specific subfamilies were associated with musical modes (see Table S2 in the Supplementary material). This test is used to determine if significant differences in proportions between paired data exist. The tests were performed using the “mcnemar.test” function from the “stats” package (R Core Team, 2024). All tests used the Bonferroni correction to account for multiple comparisons. Finally, to understand the patterns and relationships between these stimuli, cosine similarity values were calculated between the frequency distributions of different fragrance subfamilies across the inverted musical mode pairs.

### Results

**Figure 8.**
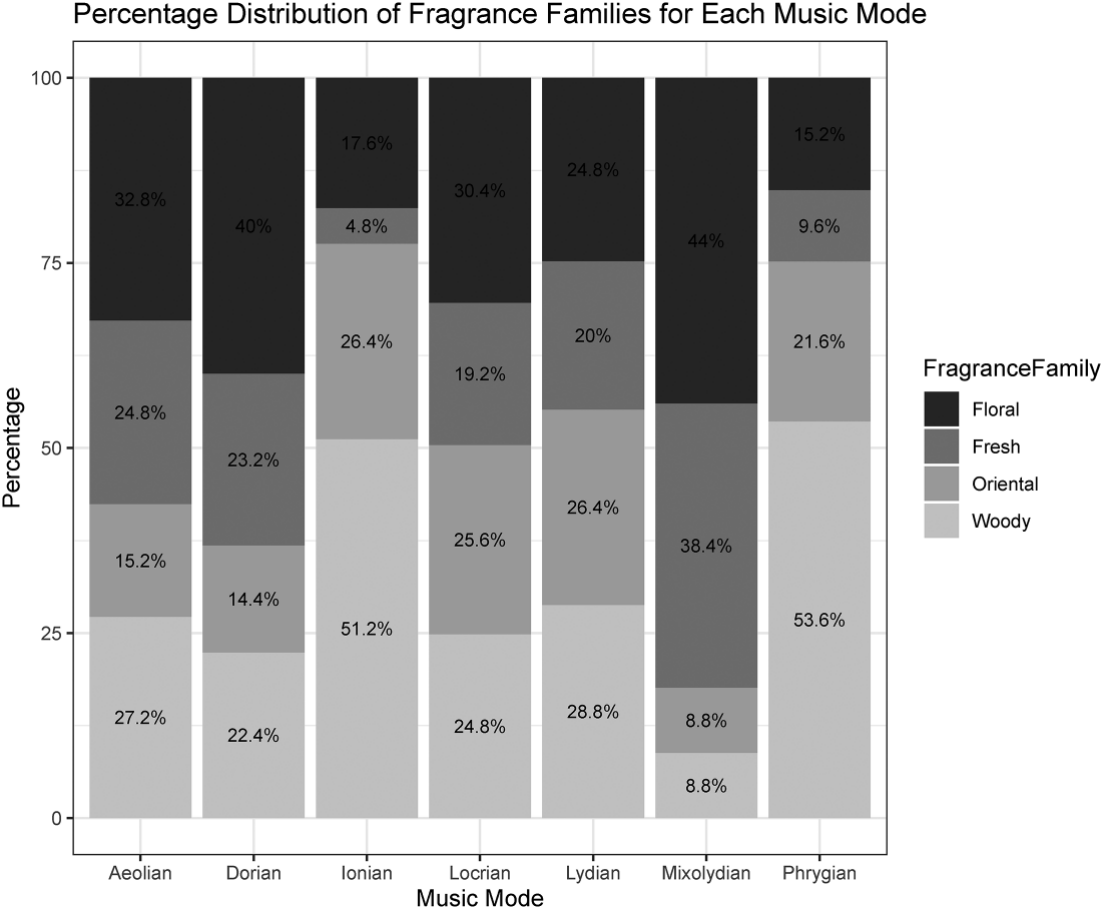
Fragrance associations with musical modes in Experiment 2: Percentage distribution of fragrance families per music mode.

*Note*. The figure illustrates the percentage distribution of four fragrance families--floral, fresh, oriental, and woody, associated with each music mode. Each vertical bar represents one mode and is divided into segments corresponding to the four fragrance families. The height of each segment indicates the proportion of participants who associated that fragrance family with the respective music mode. Percentages are labelled within each segment to aid interpretation.

First, the frequency of each fragrance family within each musical mode was calculated to understand the distribution patterns. The chi-square test results revealed a significant association between these stimuli (χ^2^(18, 125) = 155.34, *p* < .001), indicating that the distribution of fragrance families varied significantly across different musical modes (Figure 8).

Table 3 shows the results for the comparisons between the observed proportions of selected fragrance families across different musical modes. The results reveal distinct patterns of fragrance–mode associations. Specifically, participants matched the Ionian mode with the woody family at a level that was significantly above chance with a medium effect size. In contrast, they matched the Ionian mode with the fresh and floral families significantly below the chance level with large and small effect sizes, respectively. The Mixolydian mode was matched with the floral and fresh families significantly above the chance level with small-to-medium effect sizes. Conversely, it was matched with the oriental and woody families at a level that was significantly below chance and the effect sizes were medium. The Dorian mode was matched with the floral family significantly above the chance level, while it was matched with the oriental family below the chance level, both associations showing small-to-medium effect sizes. The Aeolian mode was matched with the oriental family significantly below the chance level, while it tended to be matched with the floral family significantly above the chance level, both results showing small effect sizes. Finally, participants matched the Phrygian mode with the woody family significantly above the chance level with a medium effect size. In contrast, Phrygian was matched with the fresh and floral families significantly below the chance level with medium and small effect sizes, respectively. Finally, the Lydian and Locrian modes were not significantly associated with any of the fragrance families, suggesting that these modes could be perceived as more varied and neutral.

**Table 3.** One-sample *t*-test analysis of family associations in Experiment 2.

Mode	Family	Proportion	***t***-Statistic	***p*** value	Cohen's ***d***
Lydian	Floral	24.8	−0.05	.95	
	Fresh	20.0	−1.39	.16	
	Oriental	26.4	0.35	.72	
	Woody	28.8	0.93	.35	
Ionian	Floral	17.6	−2.16	.032*	0.19
	Fresh	4.8	−10.52	<.001**	0.94
	Oriental	26.4	0.35	.72	
	Woody	51.2	5.83	<.001**	0.52
Mixolydian	Floral	44.0	4.2	<.001**	0.38
	Fresh	38.4	3.06	.002**	0.27
	Oriental	8.8	−6.36	<.001**	0.57
	Woody	8.8	−6.36	<.001**	0.57
Dorian	Floral	40.0	3.40	<.001**	0.30
	Fresh	23.2	−0.47	.63	
	Oriental	14.4	−3.36	.001**	0.30
	Woody	22.4	−0.69	.48	
Aeolian	Floral	32.8	1.85	.06	0.1655
	Fresh	24.8	−0.05	.95	
	Oriental	15.2	−3.03	.002**	0.2719
	Woody	27.2	0.55	.58	
Phrygian	Floral	15.2	−3.03	.002**	0.27
	Fresh	9.6	−5.82	<.001**	0.52
	Oriental	21.6	−0.92	.35	
	Woody	53.6	6.38	<.001**	0.57
Locrian	Floral	30.4	1.30	.19	
	Fresh	19.2	−1.63	.10	
	Oriental	25.6	0.15	.87	
	Woody	24.8	−0.05	.95	

*Note*. Overview of the statistical analyses conducted on the fragrance families and musical modes matchings. Cohen's *d* was calculated and interpreted for significant deviations from the chance level.

*p < .05, **p < .001.

Table 4 shows the results for the comparisons between the observed proportions of the musical modes across the different fragrance subfamilies. For Ionian, the woody subfamilies such as mossy woods, woody oriental, and wood showed significant associations. This indicates a strong association between the Ionian mode and woody scents. For Mixolydian, the subfamilies green, floral, and soft floral showed significant associations. The Dorian mode showed stronger associations with the floral, soft floral, and water subfamilies, while it was weakly associated with the other subfamilies. This indicates a higher association with lighter, more floral scents. The Aeolian mode showed significant associations with the mossy woods, water, and floral oriental subfamilies, reflecting the mode's thematic association with “water elements.” This suggests that Aeolian's perceptual qualities align well with water-related scents. The Phrygian mode showed stronger associations with the woods, mossy woods, and woody oriental subfamilies. For both Lydian and Locrian modes, the results were not statistically significant for any fragrance family overall.

**Table 4.** Mode subfamily proportions in Experiment 2.

Subfamily	Aeolian	Dorian	Ionian	Mixolydian	Lydian	Phrygian	Locrian
Aromatic	0.040	0.040	0.008	0.048	0.024	0.032	0.008
Citrus	0.016	0.040	0.008	0.040	0.032	0.016	0.024
Water	**0**.**128**	**0**.**104**	0.024	0.096	**0**.**096**	0.032	**0**.**096**
Green	0.064	0.048	0.008	**0**.**200**	0.048	0.016	**0**.**064**
Fruity	0.032	0.016	0.008	0.072	0.024	0.024	0.032
Soft floral	0.104	**0**.**160**	0.080	**0**.**160**	**0**.**120**	0.032	**0**.**088**
Floral	0.104	**0**.**184**	0.040	**0**.**160**	**0**.**064**	0.064	**0**.**112**
Floral oriental	**0**.**128**	0.072	0.096	0.056	**0**.**096**	0.056	**0**.**144**
Soft oriental	0.056	0.064	0.064	0.064	**0**.**120**	0.064	0.056
Oriental	0.032	0.024	0.088	0.008	0.056	0.040	**0**.**080**
Woody Oriental	0.064	0.056	**0**.**128**	0.008	**0**.**096**	**0**.**168**	**0**.**104**
Woods	0.064	0.088	**0**.**120**	0.040	**0**.**064**	**0**.**200**	**0**.**064**
Mossy Woods	**0**.**160**	0.032	**0**.**224**	0.024	**0**.**120**	**0**.**152**	**0**.**096**
Dry woods	0.008	0.072	0.104	0.024	0.040	**0**.**104**	0.032

*Note*. This table shows the proportions of matchings of each subfamily within the diatonic modes, calculated without considering previous family matches. Most significant subfamilies are highlighted in bold. Supplementary Table S2 reports the statistics from the McNemar tests using these proportions with significant results highlighted in bold.

The influence of mode inversion on fragrance associations was examined, rooted in the symmetry and properties of the diatonic system. Cosine similarity values between the frequency distributions of different fragrance subfamilies across the inverted musical mode pairs were calculated to understand the patterns and relationship between these stimuli (see Table 5). The results revealed a high cosine similarity value between the Ionian and Phrygian modes, reinforcing the notion that these modes, when inverted, share a significant overlap in fragrance subfamily distributions. Similarly, the cosine similarity value between the Lydian and Locrian modes indicates a highly comparable distribution of their associated fragrance subfamilies, supporting the theoretical symmetry of these modes upon inversion. Regarding Aeolian and Mixolydian, while both are primarily associated with floral fragrances, Aeolian also shows a higher association with water and mossy woods. The cosine similarity value suggests a moderate to high similarity, reflecting their distinct yet related matched fragrance profiles. Finally, Dorian's cosine similarity values with Aeolian and Mixolydian highlight the modes’ intermediate nature, bridging the characteristics of these two modes in terms of fragrance associations.

**Table 5.** Cosine similarity values between inverted mode pairs.

Mode pair	Cosine similarity
Ionian vs. Phrygian	0.912
Lydian vs. Locrian	0.934
Aeolian vs. Mixolydian	0.746
Aeolian vs. Dorian	0.824
Mixolydian vs. Dorian	0.847

*Note*: The cosine similarity value ranges from −1 to 1, with 1 indicating identical distributions.

### Discussion

The results of Experiment 2 demonstrated consistent associations between musical modes and fragrance families. Specifically, the Ionian mode, often linked to bright and uplifting tonal qualities, was predominantly matched with the woody fragrance family, which is characterized as more intense and darker in the first experiment. Conversely, the Mixolydian mode was strongly associated with floral, as well as the green subfamily from the fresh category. This result aligns with that of Experiment 1, where major modes, including Mixolydian, were rated as brighter and less intense, a pattern also observed for floral and fresh fragrances. Importantly, these findings partially support our hypothesis on the expected alignment between “brighter” musical modes and lighter, floral, and fresh scents. On the other hand, the Phrygian mode was primarily associated with woody fragrances and exhibited negative associations with fresh and floral scents. Given that Phrygian is often described as dark, this result suggests a perceptual link between minor, tension-inducing scales and darker fragrance profiles. Notably, the Aeolian and Dorian modes showed tendencies towards floral and water-based subfamilies.

One of the novel aspects of Experiment 2 was the analysis of mode inversions, which revealed strong cosine similarity values between inverted mode pairs. The high similarity between Ionian and Phrygian, as well as Lydian and Locrian, reinforces the idea that structurally inverted modes share perceptual commonalities in fragrance associations. These findings align with previous research in music cognition, which suggests that mode inversion retains essential structural properties that influence perception (Deutsch, 1999, 2013; Lerdahl, 2001). While some mode pairs (e.g. Ionian–Phrygian, Lydian–Locrian) showed high similarity, others such as Aeolian–Mixolydian demonstrated a more moderate resemblance. This suggests that while inversion plays a role in shaping crossmodal associations, it does not override the inherent perceptual characteristics of each mode. Additionally, the finding that Dorian serves as an intermediate mode further supports its flexible nature in auditory and olfactory mappings.

## General Discussion

This study assessed the possible association between complex auditory stimuli and olfactory imagery. Specifically, we designed two studies, one exploratory and one confirmatory, using musical modes and fragrances. The results provided empirical support for the existence of crossmodal correspondences between qualities in these two sensory modalities. Participants were presented with different sound samples of a melody converted into the seven musical modes and conceptual descriptions of fragrance families and subfamilies. In Experiment 1, the participants evaluated the stimuli in terms of liking, brightness, and intensity, and using semantic differential scales. At the same time, they also reported their thoughts and associations with each stimulus. In Experiment 2, the participants matched the modes and fragrances with the objective of examining possible crossmodal associations between these stimuli. It is important to note that traditionally, crossmodal studies tend to focus on specific pairings of individual stimuli, such as a particular sound with a particular scent. Our study, however, used broader categories, matching musical modes with fragrance families and subfamilies, neither with a seeming structured organization framework.

The results revealed consistent patterns of associations between musical modes and fragrance imagery. Specifically, the Ionian mode was predominantly associated with woody fragrances. In both the attribute ratings (SDT) and thematic analyses, the oriental and floral families are closely paired with the Ionian mode. However, the woody family, except for the strong thematic congruency with woody oriental, is not as strongly associated with the Ionian mode in those analyses. Similarly, the Phrygian mode also showed a strong association with woody fragrances as well as exhibiting weak association with fresh fragrances, consistent with the low brightness ratings. These strong associations for woody scents are reinforced by Experiment 1's thematic and attribute-based analyses, highlighting a robust alignment of the Phrygian mode with woody characteristics. The Aeolian mode was primarily matched with floral fragrances, with a notable association with the water and mossy woods subfamilies. The Mixolydian mode was highly associated with floral and fresh fragrances, while it was weakly associated to both the oriental and woody families. The Dorian mode exhibited fragrance association similar both to the Mixolydian and Aeolian, with notable associations to floral fragrances but also a balanced association with fresh and woody scents. For the Lydian and Locrian modes, the results were more nuanced. Both modes were not significantly associated to any fragrance family overall. However, detailed analysis of subfamilies revealed frequent associations of these modes within certain subfamilies. Specifically, soft floral, floral oriental, and mossy woods subfamilies within the Lydian mode, and floral oriental, floral, and woody oriental within the Locrian mode showed significant weaker associations.

To further investigate the specific associations within each mode, detailed analyses of subfamily associations revealed additional insights. Within the Aeolian mode, participants showed strong associations for the mossy woods and water subfamilies. In the Mixolydian mode, the green and soft floral subfamilies were most associated, reflecting the mode's high brightness and likability ratings and low intensity ratings. Despite previous thematic analysis suggesting an association with the oriental subfamily, the current analysis indicates a stronger alignment with floral and fresh attributes.

### How and Why is it that People Associate Musical Modes and Olfactory Imagery?

The association between musical modes and olfactory imagery can be attributed to several complementary mechanisms underlying crossmodal correspondences. Statistical correspondences are related to the internalization of the statistical regularities of the environment established based on experience. The structural correspondences refer to those neural systems we use to code sensory information and the semantic correspondences are those based on a common identity or meaning (Motoki et al., 2024; Spence, 2011). It is important to note that these mechanisms may not be mutually exclusive and can work together in a hierarchical manner.

The high cosine similarity values between inverted mode pairs support the idea that structural symmetry plays a key role in these associations. Specifically, while mirror inversion is a common transformation in music, it is not generally perceived as symmetrical (Bianchi et al., 2017), and previous studies indicate that mirror modes differ in affective valence, brightness, and semantic content (Ramos et al., 2011). Nonetheless, our results suggest that fragrance associations exhibit a structured relationship when mapped onto these inverted modes, as captured by cosine similarity measures. This unexpected finding suggests that theoretical symmetry at a structural level may contribute to these associations, even when perceptual symmetry in the musical domain is weak or absent.

Brightness, a putatively amodal sensory dimension (though see Spence & Di Stefano, 2024), as a potential mediating factor, may influence these associations as seen in the first study. Major modes, considered brighter, tended to have a higher proportion of floral and fresh fragrances, while minor modes were more aligned with woody and oriental fragrances. However, the absence of significant differences in Experiment 2 may suggest that brightness alone does not fully account for the observed correspondences, as the expected associations between major modes (floral and fresh) and minor modes (woody and oriental) did not emerge consistently in the matching task (though note that the absence of evidence is not evidence of absence in the statistical approach used in the present research).

Similarly, intensity and liking may also play a role in these associations but do not fully account for the observed patterns. While Locrian was perceived as the most intense mode and Mixolydian and Lydian were the most liked, these distinctions were not consistently reflected in the fragrance–music pairings. Likewise, although certain fragrance families, such as woody oriental, were rated as more intense and fruity as more liked, these attributes did not directly predict their associations with specific musical modes. Additionally, connotative meaning and semantic congruence also play a significant role in some associations. The Mixolydian mode, for instance, was highly associated with the green subfamily, as seen in the SDT analysis. Similarly, the Aeolian mode showed strong associations based on the SDT analysis of attribute ratings, being highly matched to mossy woods and the water subfamily. This aligns with the growing recognition that semantic factors significantly contribute to how we perceive and integrate multisensory information (Alvarado et al., 2024). The suggested mechanisms behind the observed correspondences are only possibilities and further research is needed to confirm them. As an exploratory study, the present research aimed to identify potential patterns and relationships between auditory and olfactory stimuli. Overall, the results provide evidence to the idea that the way we perceive and categorize these sensory stimuli is influenced by both intrinsic properties of the stimuli and the semantic meanings assigned to them. Research summarized by Spence (2020b) highlights a surge in interest in crossmodal correspondences, particularly those involving auditory (musical) stimuli. Similarly, Walker (2016) provides a theoretical framework illustrating the cross-sensory correspondences between basic features of bodily actions, gestures, and vocalizations, and the perception of musical sounds. Our study contributes to the relatively unexplored area of complex stimuli by demonstrating that musical modes are consistently associated with specific fragrance imagery.

### Limitations and Future Research

The present study does not come without limitations. For example, not all participants showed the same patterns of matches, indicating high variability in individual responses. While there was significant agreement in many matches, the heterogeneity highlights that these correspondences are not universal. Future research should explore the factors that contribute to these differences, such as the familiarity with the different stimuli or the sensitivity that each participant shows to each of these particular correspondences (Chuquichambi et al., 2024). Another limitation is the use of fragrance imagery rather than actual fragrances, which introduces a level of abstraction and interpretation. Participants’ perceptions of fragrance imagery might differ from their reactions to real scents, potentially affecting the accuracy of the matches. Future research should incorporate actual olfactory stimuli to provide a more realistic assessment of crossmodal correspondences and reduce the abstraction level.

Another potential limitation concerns the available sample size in relation to the number of factor levels. While prior research in crossmodal correspondences involving audition and olfaction (e.g. Crisinel et al., 2013; Crisinel & Spence, 2012a) successfully identified significant associations with smaller sample sizes, those studies used real olfactory stimuli, whereas the present study relies on the mental imagery for fragrance categories. This methodological difference, coupled with the larger and more complex stimulus set used here (musical modes and fragrance families), may introduce additional variability in responses. However, the within-subjects design of the study ensured that each participant contributed to all the conditions, and the number of factor levels (7 for musical modes, 4 and 14 for fragrance families and subfamilies) reflects the inherent structure of the stimuli. Future research using musical modes and fragrances would benefit from larger participant samples and the inclusion of complementary methodological approaches.

Furthermore, we only used a basic sound check to assess participants’ hearing capabilities, which may be insufficient for older adults or those with mild impairment. Likewise, standardized attention checks were not implemented and the online nature of the experiments (e.g. volume settings, device types, and ambient noise conditions) could have influenced participants’ auditory experiences and subsequent evaluations. However, despite these limitations, research supports low levels of attentional disengagement and an acceptable variability in data quality in online tasks compared to standards for lab-based testing (Albert & Smilek, 2023; Uittenhove et al., 2023).

Finally, this study diverges from traditional crossmodal research, which often focuses on specific and direct pairings of stimuli, by exploring broader categories such as musical modes and fragrance families. The results support the suggestion that our brains can associate complex and abstract sensory information across modalities, aligning with emerging research on multisensory integration. Our results also have implications for sensory marketing, for example, matching specific music with scents to create more engaging, semantic, and emotionally congruent experiences for consumers (e.g. Spence et al., 2024). Future studies could focus on evaluating the contexts under which different mechanisms are used and the extent to which they guide the matches. This approach will help to clarify the hierarchical nature of these mechanisms and their interactions.

## Supplemental Material

sj-docx-1-pec-10.1177_03010066251342011 - Supplemental material for The diatonic sound of scent imagery Supplemental material, sj-docx-1-pec-10.1177_03010066251342011 for The diatonic sound of scent imagery by Oriente Pimentel, Erick G. Chuquichambi, Charles Spence and Carlos Velasco in Perception
