# Supplementary material for: The diatonic sound of scent imagery
Source: Perception. 2025 Jun 3;54(9):689–714. doi: 10.1177/03010066251342011 (PMC12326031; doi:10.1177/03010066251342011)
Supplement: sj-docx-1-pec-10.1177_03010066251342011 - Supplemental material for The diatonic sound of scent imagery [file sj-docx-1-pec-10.1177_03010066251342011.docx]

**Supplementary materials**

**Table S1.** Descriptive statistics for likeability, brightness, and intensity across fragrance families and musical modes in Experiment 1.

| Category | Task | Stimulus | Mean range | SD Range | SE Range |
| --- | --- | --- | --- | --- | --- |
| Family | Liking | Floral | 6.94 | 2.23 | 11.11 |
|  |  | Fresh | 6.75 | 2.34 | 11.66 |
|  |  | Oriental | 6.56 | 2.30 | 13.20 |
|  |  | Woody | 6.14 | 2.55 | 14.66 |
|  | Brightness | Floral | 6.97 | 1.99 | 9.88 |
|  |  | Fresh | 6.66 | 2.26 | 11.22 |
|  |  | Oriental | 4.96 | 2.12 | 12.18 |
|  |  | Woody | 4.10 | 2.23 | 12.81 |
|  | Intensity | Floral | 5.81 | 2.06 | 10.24 |
|  |  | Fresh | 5.83 | 2.32 | 11.55 |
|  |  | Oriental | 6.57 | 1.94 | 11.15 |
|  |  | Woody | 6.53 | 1.91 | 10.98 |
| Musical mode | Liking | Aeolian | 6.89 | 2.12 | 2.11 |
|  |  | Dorian | 6.53 | 2.14 | 2.13 |
|  |  | Ionian | 6.78 | 2.13 | 2.12 |
|  |  | Locrian | 5.72 | 2.65 | 2.64 |
|  |  | Lydian | 7.13 | 1.87 | 1.86 |
|  |  | Mixolydian | 7.22 | 1.74 | 1.73 |
|  |  | Phrygian | 6.42 | 2.05 | 2.04 |
|  | Brightness | Aeolian | 5.21 | 2.12 | 2.11 |
|  |  | Dorian | 4.45 | 1.77 | 1.77 |
|  |  | Ionian | 6.02 | 2.03 | 2.02 |
|  |  | Locrian | 3.57 | 1.81 | 1.80 |
|  |  | Lydian | 6.36 | 2.13 | 2.12 |
|  |  | Mixolydian | 7.00 | 1.97 | 1.97 |
|  |  | Phrygian | 5.01 | 1.99 | 1.98 |
|  | Intensity | Aeolian | 5.30 | 2.09 | 2.08 |
|  |  | Dorian | 5.60 | 1.73 | 1.72 |
|  |  | Ionian | 4.40 | 1.79 | 1.78 |
|  |  | Locrian | 6.57 | 1.61 | 1.60 |
|  |  | Lydian | 4.83 | 1.76 | 1.75 |
|  |  | Mixolydian | 4.70 | 2.06 | 2.05 |
|  |  | Phrygian | 5.09 | 1.93 | 1.92 |

*Note. Descriptive statistics (mean, standard deviation, and standard error) for three different dimensions (likeability, brightness, intensity) across the fragrance families and musical modes.*

**Table S2.** McNemar Test Results for Experiment 2.

| Musical mode | | Aeolian | Dorian | Ionian | Locrian | Lydian | Mixolydian | Phrygian |
| --- | --- | --- | --- | --- | --- | --- | --- | --- |
| Aromatic | Citrus  Water  Green  Fruity  Soft Floral  Floral  Floral Oriental  Soft Oriental  Oriental  Oriental Woody  Woods  Mossy Woods  Dry Woods | 0.57  **4.76**  0.31  0.00  2.72  2.72  **4.76**  0.08  0.00  0.31  0.31  **7.84**  1.50 | 0.00  2.72  0.00  0.57  **7.84**  **10.32**  0.64  0.31  0.13  0.08  1.56  0.00  0.64 | 0.00  0.25  0.00  0.00  **5.82**  1.50  **7.69**  **4.00**  **6.75**  **11.53**  **10.56**  **23.21**  **8.64** | 0.25  **7.69**  **4.00**  0.80  **6.75**  **9.60**  **13.47**  3.13  **5.82**  **8.64**  **4.00**  **7.69**  0.80 | 0.00  **4.27**  0.45  0.00  **6.72**  1.45  **4.27**  **6.72**  0.90  **4.27**  1.45  **6.72**  0.13 | 0.00  1.39  **10.45**  0.27  **6.5**  **6.5**  0.00  0.07  2.29  2.29  0.00  0.44  0.44 | 0.17  0.00  0.17  0.00  0.00  0.75  0.36  0.75  0.00  **10.24**  **13.79**  **8.52**  3.76 |
| Citrus | Water  Green  Fruity  Soft Floral  Floral  Floral Oriental  Soft Oriental  Oriental  Oriental Woody  Woods  Mossy Woods  Dry Woods | **9.39**  2.50  0.17  **6.67**  **6.67**  **9.39**  1.78  0.17  2.50  2.50  **13.14**  0.00 | 2.72  0.00  0.57  **7.84**  **10.32**  0.64  0.31  0.13  0.08  1.56  0.00  0.64 | 0.25  0.00  0.00  **5.82**  1.50  **7.69**  **4.00**  **6.75**  **11.53**  **10.56**  **23.21**  **8.64** | **4.27**  1.45  0.00  3.50  **5.88**  **9.33**  0.90  2.77  **5.06**  1.45  **4.27**  0.00 | 3.06  0.10  0.00  **5.26**  0.75  3.06  **5.26**  0.36  3.06  0.75  **5.26**  0.00 | 2.12  **12.03**  0.64  **7.84**  **7.84**  0.08  0.31  1.50  1.50  0.00  0.13  0.13 | 0.17  0.00  0.00  0.17  2.50  1.78  2.50  0.57  **14.09**  **17.93**  **12.19**  **6.67** |
| Water | Green  Fruity  Soft Floral  Floral  Floral Oriental  Soft Oriental  Oriental  Oriental Woody  Woods  Mossy Woods  Dry Woods | 2.04  **6.05**  0.14  0.14  0.00  2.78  **6.05**  2.04  2.04  0.25  **11.53** | 1.89  **6.67**  1.09  2.25  0.41  0.76  **5.06**  1.25  0.04  3.76  0.41 | 0.25  0.25  2.77  0.13  **4.27**  1.45  3.50  **7.58**  **6.72**  **18.58**  **5.06** | 0.45  3.06  0.00  0.04  0.83  0.84  0.05  0.00  0.45  0.00  3.06 | 1.39  **4.27**  0.15  0.45  0.00  0.15  0.84  0.00  0.45  0.15  2.12 | 3.89  0.19  1.53  1.53  0.84  0.45  **7.69**  **7.69**  2.12  **4.27**  **4.27** | 0.17  0.00  0.00  00.75  0.36  0.75  0.00  **10.24**  **13.79**  **8.52**  3.76 |
| Green | Fruity  Soft Floral  Floral  Floral Oriental  Soft Oriental  Oriental  Oriental Woody  Woods  Mossy Woods  Dry Woods | 0.75  0.76  0.76  2.04  0.00  0.75  0.00  0.00  **4.32**  **4.00** | 1.13  **6.50**  **8.83**  0.27  0.07  0.44  0.00  0.94  0.1  0.27 | **0.00**  **5.82**  1.50  **7.69**  **4.00**  **6.75**  **11.53**  **10.56**  **23.31**  **8.64** | 0.75  0.21  1.14  3.13  0.00  0.06  0.76  0.00  0.45  0.75 | 0.44  3.05  0.07  1.39  3.05  0.00  1.39  0.07  3.05  0.00 | **6.62**  0.36  0.36  **9.03**  **7.76**  **20.35**  **20.35**  **12.03**  **15.75**  **15.75** | 0.00  0.17  2.50  1.78  2.50  0.57  **14.09**  **17.93**  **12.19**  **6.67** |
| Fruity | Soft Floral  Floral  Floral Oriental  Soft Oriental  Oriental  Oriental Woody  Woods  Mossy Woods  Dry Woods | 3.76  3.76  **6.05**  0.36  0.00  0.75  0.75  **9.37**  0.80 | **13.14**  **16.00**  3.27  2.50  0.00  1.78  **4.92**  0.17  3.27 | **5.82**  1.50  **7.69**  **4.00**  **6.75**  **11.53**  **10.56**  **23.31**  **8.64** | 2.40  **4.50**  **7.68**  0.36  1.79  3.76  0.75  3.06  0.00 | **6.72**  1.45  **4.27**  **6.72**  0.90  **4.27**  1.45  **6.72**  0.13 | 3.45  3.45  0.06  0.00  **4.90**  **4.90**  0.64  2.08  2.08 | 0.00  1.45  0.90  1.45  0.13  **12.04**  **15.75**  **10.23**  **5.06** |
| Soft Floral | Floral  Floral Oriental  Soft Oriental  Oriental  Oriental Woody  Woods  Mossy Woods  Dry Woods | 0.00  0.13  1.25  3.76  0.76  0.76  1.09  **8.64** | 0.09  3.45  **4.32**  **11.13**  **5.33**  2.06  **9.38**  3.45 | 1.07  0.05  0.06  0.00  0.96  0.64  **7.61**  0.17 | 0.16  1.24  0.50  0.00  0.04  0.21  0.00  2.40 | 1.57  0.15  0.00  2.23  0.15  1.57  0.00  **4.05** | 0.00  5.33  4.32  15.43  15.43  7.84  11.13  11.13 | 0.75  0.36  0.75  0.00  **10.24**  **13.79**  **8.52**  3.76 |
| Floral | Floral Oriental  Soft Oriental  Oriental  Oriental Woody  Woods  Mossy Woods  Dry Woods | 0.14  1.25  3.76  0.76  0.76  1.09  **8.64** | **5.28**  **6.32**  **13.88**  **7.50**  3.56  **12.00**  **5.28** | 2.12  0.31  1.56  **4.76**  **4.05**  **14.67**  2.72 | 0.28  1.71  0.38  0.00  1.14  0.04  **4.50** | 0.45  1.57  0.00  0.45  0.00  1.57  0.31 | 5.33  4.32  15.43  15.43  7.84  11.13  11.13 | 0.00  0.00  0.31  **4.97**  **7.76**  3.70  0.76 |
| Floral Oriental | Soft Oriental  Oriental  Oriental Woody  Woods  Mossy Woods  Dry Woods | 2.78  **6.05**  2.04  2.04  0.25  **11.52** | 0.00  2.08  0.06  0.05  1.23  0.00 | 0.45  0.00  0.32  0.15  **5.63**  0.00 | **4.00**  1.75  0.52  3.12  0.83  **7.68** | 0.15  0.84  0.00  0.45  0.15  2.12 | 0.00  3.13  3.13  0.08  0.90  0.90 | 0.00  0.08  **6.04**  **9.03**  **4.65**  1.25 |
| Soft Oriental | Oriental  Oriental Woody  Woods  Mossy Woods  Dry Woods | 0.36  0.00  0.00  **5.33**  3.13 | 1.45  0.00  0.21  0.75  0.00 | 0.21  2.04  1.57  **10.03**  0.76 | 0.24  1.25  0.00  0.84  0.36 | 2.23  0.15  1.57  0.00  **4.05** | 4.00  4.00  0.31  1.45  1.45 | 0.31  **4.97**  **7.76**  3.70  0.76 |
| Oriental | Oriental Woody  Woods  Mossy Woods  Dry Woods | 0.75  0.75  **9.38**  0.80 | 0.90  3.50  0.00  2.08 | 0.59  0.35  **6.56**  0.04 | 0.17  0.06  0.05  1.79 | 0.84  0.00  2.23  0.08 | 0.00  1.50  0.25  0.25 | **8.65**  **12.03**  **7.04**  2.72 |
| Woody Oriental | Woods  Mossy Woods  Dry Woods | 0.00  **4.32**  **4.00** | 0.50  0.36  0.06 | 0.00  2.75  0.14 | 0.76  0.00  3.76 | 0.45  0.15  2.12 | 1.50  0.25  0.25 | 0.20  0.03  1.44 |
| Woods | Mossy Woods  Dry Woods | **4.32**  **4.00** | 2.40  0.05 | 3.35  0.04 | 0.45  0.75 | 1.57  0.31 | 0.13  0.13 | 0.57  3.18 |
| Mossy Woods | Dry Woods | **15.42** | 1.23 | **4.78** | 3.06 | **4.05** | 0.00 | 0.78 |

*Note. Values represent Chi-Square (𝜒2) statistics from the McNemar tests in Experiment 2. Significant differences (p < 0.05) are highlighted in bold.*

**Figure S1.** Heatmap of attribute ratings across fragrance subfamilies and musical modes in Experiment 1.


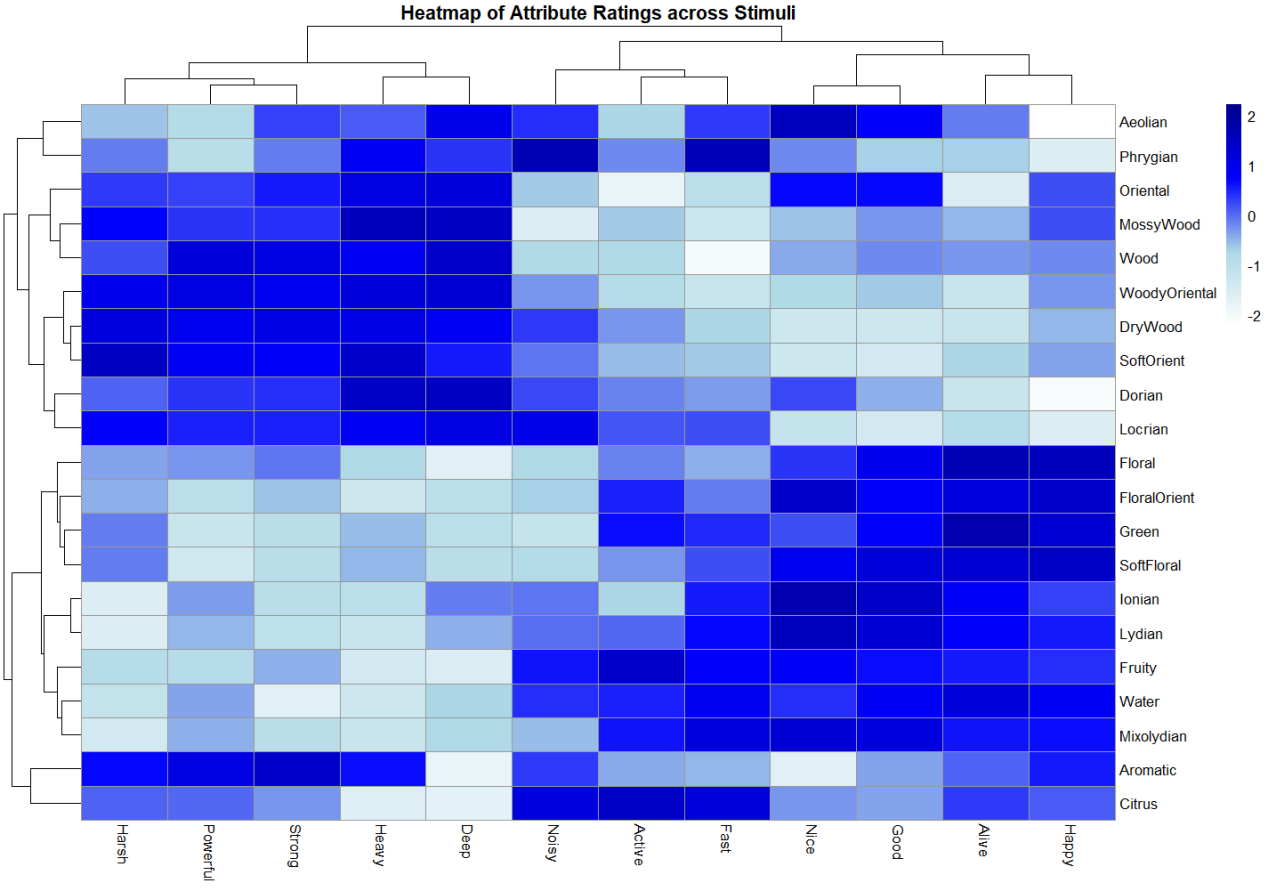


*Note. Hierarchical clustering was performed to group similar stimuli and attributes based on their ratings. Dark blue indicates higher ratings for the attribute, suggesting that the stimulus is strongly associated with the attribute, whereas lighter colours indicate lower associations.*

**Figure S2.** Heatmap of theme presence across fragrances and modes in Experiment 1.


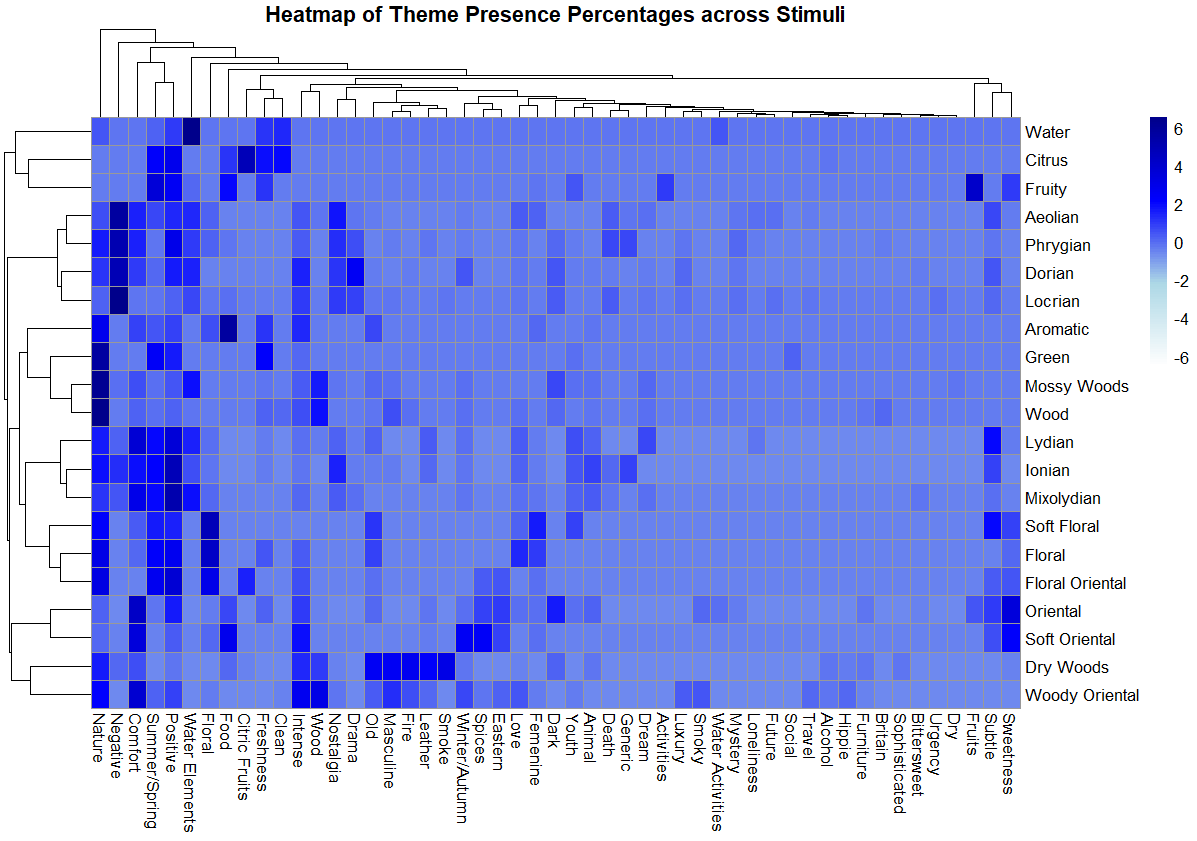


*Note. The clustered heatmap provides a visual representation of the similarities between different musical modes and fragrance families based on the frequency of themes mentioned for each. Colour intensity represents the degree of similarity between the musical modes and fragrance families. Darker blues suggest higher similarity, while lighter colours suggest lower similarity.*
